# Design, Physicochemical Characterization, and Antibacterial Activity of Freeze‐Dried Sodium Alginate Sponges Loaded With Amazonian Oil (*Carapa guianensis* Aubl.) and a Mn(II)‐Phenanthroline Complex for Potential Use in Topical Applications

**DOI:** 10.1002/cbdv.202502812

**Published:** 2026-07-15

**Authors:** Marinaldo V. de Souza Junior, João G. de Oliveira Neto, Jad L. F. Simplicio, Djany S. Silva, Jaqueline D. S. Barros, Richard P. Dutra, Eliana B. Souto, Adenilson O. dos Santos, Francisco F. de Sousa

**Affiliations:** ^1^ Center Sciences of Imperatriz Federal University of Maranhão – UFMA Imperatriz Maranhao Brazil; ^2^ UCD School of Chemical and Bioprocess Engineering University College Dublin Dublin Ireland; ^3^ Laboratory of Natural Products Chemistry Federal University of Maranhão – UFMA Imperatriz Brazil; ^4^ Institute of Exact and Natural Sciences Federal University of Para – UFPA Belém Para Brazil

**Keywords:** andiroba oil, antibacterial properties, Mn(II)‐complex, sodium‐alginate sponges, topical scaffolds

## Abstract

This study reports the development of freeze‐dried sodium alginate sponges containing Amazonian *andiroba* oil (*Carapa guianensis* Aubl.) and [Mn(phen)_2_Cl_2_], aiming to obtain porous biomaterials with antibacterial activity for potential topical wound applications. X‐Ray diffraction revealed predominantly amorphous structures for all sponges (AA1, AA3 and AA5 containing respectively, 1, 3 and 5% (m/m) of the complex). Scanning electron microscopy showed that the unloaded alginate sponge exhibited the highest apparent surface porosity (67.200%), whereas the loaded sponges showed lower surface pore area fractions (24.470% for AA1, 36.342% for AA3, and 33.028% for AA5) and a more irregular lamellar morphology. Fourier‐transform infrared spectroscopy confirmed the characteristic functional groups of alginate and *andiroba* oil, including hydroxyl, carbonyl, and carboxylate‐related bands. Thermal analyses indicated an initial dehydration event in the range of 310–355 K, followed by decomposition between 498 and 534 K. Antibacterial activity, evaluated by disk diffusion against *Staphylococcus aureus*, *Enterococcus faecalis*, *Escherichia coli*, and *Pseudomonas aeruginosa*, increased with increasing Mn(II) complex loading. The AA3 and AA5 formulations showed the highest inhibition zones, reaching 19.50 and 24.03 mm/mg against *E. coli*, 12.38 and 16.11 mm/mg against *P. aeruginosa*, 12.62 and 15.14 mm/mg against *E. faecalis*, and 11.97 and 12.43 mm/mg against *S. aureus*, respectively. These findings demonstrate that the developed sponges are promising antibacterial alginate‐based biomaterials.

## 1 Introduction

Excessive exudation, microbial contamination, and prolonged inflammation are major challenges in the management of topical wounds and skin lesions [1, 2]. In this context, porous biomaterials capable of absorbing fluids while simultaneously serving as carriers for bioactive compounds have attracted increasing interest as multifunctional wound‐dressing platforms [3, 4, 5, 6]. Among these systems, freeze‐dried polymeric sponges are particularly promising because they combine low density, high porosity, and the ability to incorporate natural and synthetic actives into a three‐dimensional (3D) matrix [6, 7, 8]. These features make them attractive for localized treatment, especially when antibacterial and anti‐inflammatory effects are desirable [9, 10, 11, 12, 13, 14, 15, 16].

Freeze‐drying is a dehydration technique that preserves the properties of drugs and dosage forms, extending their shelf life [17, 18]. With its porous and highly absorbent structure, a freeze‐dried sponge allows for incorporation of compounds into its polymeric matrix [8]. This material can act as a controlled release platform, gradually releasing drugs to the skin or even into deeper internal tissues. Moreover, freeze‐drying ensures that drugs remain stable and active for a longer time, thereby enhancing their therapeutic efficacy [6, 7].

Sodium alginate is a natural polysaccharide mainly obtained from brown algae and has been widely explored in biomedical and pharmaceutical applications due to its biocompatibility, biodegradability, hydrophilicity, and ability to form highly porous sponge‐like structures after freeze‐drying [19, 20, 21]. In wound‐related applications, alginate is especially attractive because it helps maintain a moist environment favorable to tissue repair, can absorb exudate efficiently, and may also contribute to topical hemostatic management [21, 22, 23, 24]. For these reasons, alginate‐based sponges have received considerable attention as matrices, e.g., for wound healing and tissue engineering [21, 23, 24, 25].

In addition to the structural role of the polymeric matrix, the incorporation of bioactive compounds may offer additional functionality to alginate sponges. Antibacterial agents are relevant for preventing or reducing microbial contamination in wounds, whereas anti‐inflammatory compounds may help modulate the local inflammatory response and promote a more favorable healing environment [9, 10, 11, 15, 16]. In this sense, the association of natural oils and metal‐based complexes with alginate matrices represents an interesting strategy for obtaining multifunctional biomaterials with complementary biological properties [10, 11, 14].

Sun et al. (2024) [10] developed a chitosan‐based gel sponge with adhesive properties that enabled efficient and painless hemostatic control and prevented wound infections. The sponge was made of chitosan derivative modified with tris(hydroxymethyl) glycine, designed to have a desired multi‐hollow structure, and specific antibacterial and biocompatibility profiles. The multifunctional properties, including rapid hemostasis, enhanced wound closure, and antibacterial effects, highlight the tris(hydroxymethyl) glycine sponge as a promising solution in the medical field for wound repair and acute bleeding control.

Sultana et al. (2021) [26] obtained freeze‐dried sponges combining nanocellulose and chitosan, with the addition of antibacterial lawsone (hennotannic acid) for controlled release during wound healing. The results indicated that the advanced skin substitute has hemostatic and antibacterial properties, being beneficial for palliative care in the healing of skin wounds. Huang et al. (2017) [27] evaluated different hydrophobically‐modified chitosan sponges that self‐assemble, to determine their effectiveness in the hemostasis of the femoral artery of rats. Their results showed a hemostasis time of 86 ± 5 s, which was significantly higher than that observed with pure chitosan.

Neto et al. (2017) [23] evaluated the physicochemical properties of alginate‐gelatin sponges containing usnic acid, showing a uniform macroscopic structure, with small color variations. Microstructural observation by scanning electron microscopy (SEM) revealed a homogeneous structure with regular pores. Thermal analysis indicated the interaction between usnic acid and the biopolymer used, since the melting point of usnic acid was not detected by differential scanning calorimetry (DSC) analysis. Thermogravimetric analysis (TG) curves of the sponges exhibited similar thermal behavior, suggesting that the stability of the sponges was not affected by the presence of usnic acid. Additionally, sponges demonstrated antibiotic, anti‐inflammatory, and healing properties for dermal burns.

*Andiroba* oil (*Carapa guianensis* Aubl.), an Amazonian natural product, is recognized for its anti‐inflammatory, analgesic, and healing properties, mainly attributed to its limonoids, terpenes, and unsaturated fatty acids [28, 29]. A recent study [30] showed that sodium alginate sponges loaded with *andiroba* oil significantly reduced nitric oxide production in LPS‐activated RAW 264.7 macrophages at 250 and 500 µg/mL, while maintaining cell viability above 90% across 50–500 µg/mL, reinforcing the relevance of this oil for topical biomaterials. On the other hand, Mn(II)‐phenanthroline complexes have demonstrated antibacterial activity against *Staphylococcus aureus*, *Enterococcus faecalis*, *Escherichia coli*, and *Pseudomonas aeruginosa*, with minimum inhibitory concentration (MIC) values of 125, 15.12, 7.81, and 31.25 µg/mL, respectively [31]. In this context, the association of *andiroba* oil with a Mn(II)‐based complex in an alginate sponge may enhance the biological contribution, combining the anti‐inflammatory functionality of the oil with the antibacterial action of the metal complex.

In the present study, we used sodium alginate to produce freeze‐dried sponges, containing [Mn(phen)_2_Cl_2_] complex and *andiroba* oil, to exploit the antibacterial properties of these bioactives. After freeze‐drying, which creates a 3D porous scaffold, the sponge was molded as a matrix for topical applications. Our aim was to produce a sponge containing a crystalline complex and *andiroba* oil, and to evaluate its physicochemical and bactericidal properties and confirm that our newly developed platform  has the potential to revolutionize the treatment of skin infection and inflammation, with improved properties as a hemostatic material.

## 2 Materials and Methods

### 2.1 Materials

Sodium alginate was purchased from Sigma–Aldrich (St. Louis, MO, USA) and *andiroba* oil (*C. guianensis Aubl*.) obtained from a local market (Vigia, PA, Brazil), registered in SisGen with number A05C495. [Mn(phen)_2_Cl_2_] complex was obtained by the slow evaporation method, as described by de Souza Junior et al. (2024) [31]. For that, 2 mmol of 1,10‐phenanthroline (Biosynth, Staad, Switzerland, purity 99%) and 1 mmol of manganese chloride (Sigma–Aldrich, purity 98%) were homogenized in a mixed solution of 20 mL of methanol and 10 mL of ultrapure water, for 3 h at 360 rpm at a controlled temperature of 35°C. If not otherwise stated, ultrapure MilliQ water (Merck Millipore, Burlington, MA, USA), home supplied, was used in all experiments. The corresponding CIF has been deposited at the Cambridge Crystallographic Data Center (CCDC) under number 2213123. The structural data and geometric parameters can be accessed free of charge at https://www.ccdc.cam.ac.uk/structures/.

### 2.2 Synthesis of Sponges

Accurately weighed 1 g of sodium alginate was dissolved in 50 mL of ultrapure MilliQ water under constant magnetic stirring (320 rpm) until solubilization. After preparing this solution, the [Mn(phen)_2_Cl_2_] complex was added at different concentrations (1%, 3%, or 5% with respect to the mass of sodium alginate), followed by the addition of 1 mL of *andiroba* oil, and further mixed under constant stirring (320 rpm) for 24 h. The obtained mixture was placed in Petri dishes and frozen for 24 h. After freezing, the mixtures were freeze‐dried using a TERRONI LS3000 Freeze Dryer (Lyotech, São Carlos, Brazil) at −40°C for another 24 h.

### 2.3 Characterization Techniques

The diffractograms of the sponges were obtained by X‐Ray diffraction (XRD) measurements in an Empyrean diffractometer (Malvern PANalytical, Malvern, UK), using CuKα_1_ radiation (λ = 1.5418 Å), operating at 40 kV/40 mA. XRD pattern was collected from 5 to 50° (2θ) with a step size of 0.02° and a counting time of 2 s.

To characterize their morphology, the sponges’ micrographs were obtained using a Tescan scanning electron microscope (Vega3 SB, Kohoutovice, Czech Republic). Then, sponges were placed on carbon tapes, and a thin (20 nm) gold/palladium film was deposited by sputtering. To avoid damaging the sponges with the electron beam, the sponges were characterized by SEM with low acceleration energy (6 and 10 kV) and low electron beam intensity. The micrographs were obtained at different scales and magnifications to achieve high‐quality images.

Fourier‐transform infrared  spectra of the sponges were measured in transmission mode using the Vertex 70 V Bruker spectrometer (Bruker, Billerica, MA, USA) in the spectral range from 400 to 4000 cm^−1^. A Total Reflectance Attenuated (ATR) module A225/Q Platinum and a wide RT‐DLA TGS detector with a 6 mm aperture were used in the sample, allowing measurements above 400 cm^−1^ with a spectral resolution of 4 cm^−1^ for 100 scans.

The TG and DTA curves were performed simultaneously, with a single heating cycle in a Shimadzu thermal analyzer (DTG‐60 model, Kyoto, Japan) in the temperature range between 300 and 1150 K, using platinum crucibles with ∼4 mg of sponges, under a nitrogen atmosphere (100 mL/min) and a heating rate of 10 K/min. DSC curves were obtained using Shimadzu DSC‐60 (Kyoto, Japan) with a heating flow rate of 5 K/min, an inert nitrogen atmosphere gas flow rate of 100 mL/min, and a temperature range of 300–500 K.

### 2.4 Antibacterial Tests

The disc diffusion method was used to evaluate the antibiotic susceptibility of the sponges loaded with the [Mn(phen)_2_Cl_2_] complex and the *andiroba* oil. Four bacterial strains were used in this study: two Gram‐positive (*S. aureus* (ATCC 6538) and *E. faecalis* (ATCC 29212)) and two Gram‐negative (*E. coli* (ATCC 25922) and *P. aeruginosa* (ATCC 27853)), all obtained from American Type Culture Collection (ATCC, Manassas, VA, USA). Strains were suspended in Mueller–Hinton broth (Kasvi, MG, Brazil), and inocula were prepared following the turbidity scale corresponding to bacterial growth in Mueller–Hinton broth of 1 × 10^8^ UFC/mL compatible with a turbidity of 0.5 on the standard McFarland scale. Absorbance was obtained on a microplate spectrophotometer (BioTek Instruments, Winooski, VT, USA) in the range of 0.08–0.10 at 630 nm. The sponges were cut into 10 mm discs and placed on a plate containing Mueller–Hinton broth and incubated for 24 h at 35°C. The sponges were prepared in triplicate. The inhibition zone created by the sponges on the surface of the plate was interpreted as the antibacterial effect and compared to commercially available antibiotic susceptibility discs of gentamicin (Gibco, Waltham, MA, USA) placed in the center of each Mueller‐Hinton plate and used as a positive control.

### 2.5 Statistical Analysis

Descriptive statistics was used for bactericidal activity data, expressed as mean ± standard deviation (*n *= 3).

## 3 Results and Discussion

### 3.1 XRD and SEM Studies of Built‐in Sponges With [Mn(Phen)_2_Cl_2_] Complex and *Andiroba* Oil

**FIGURE 1:**
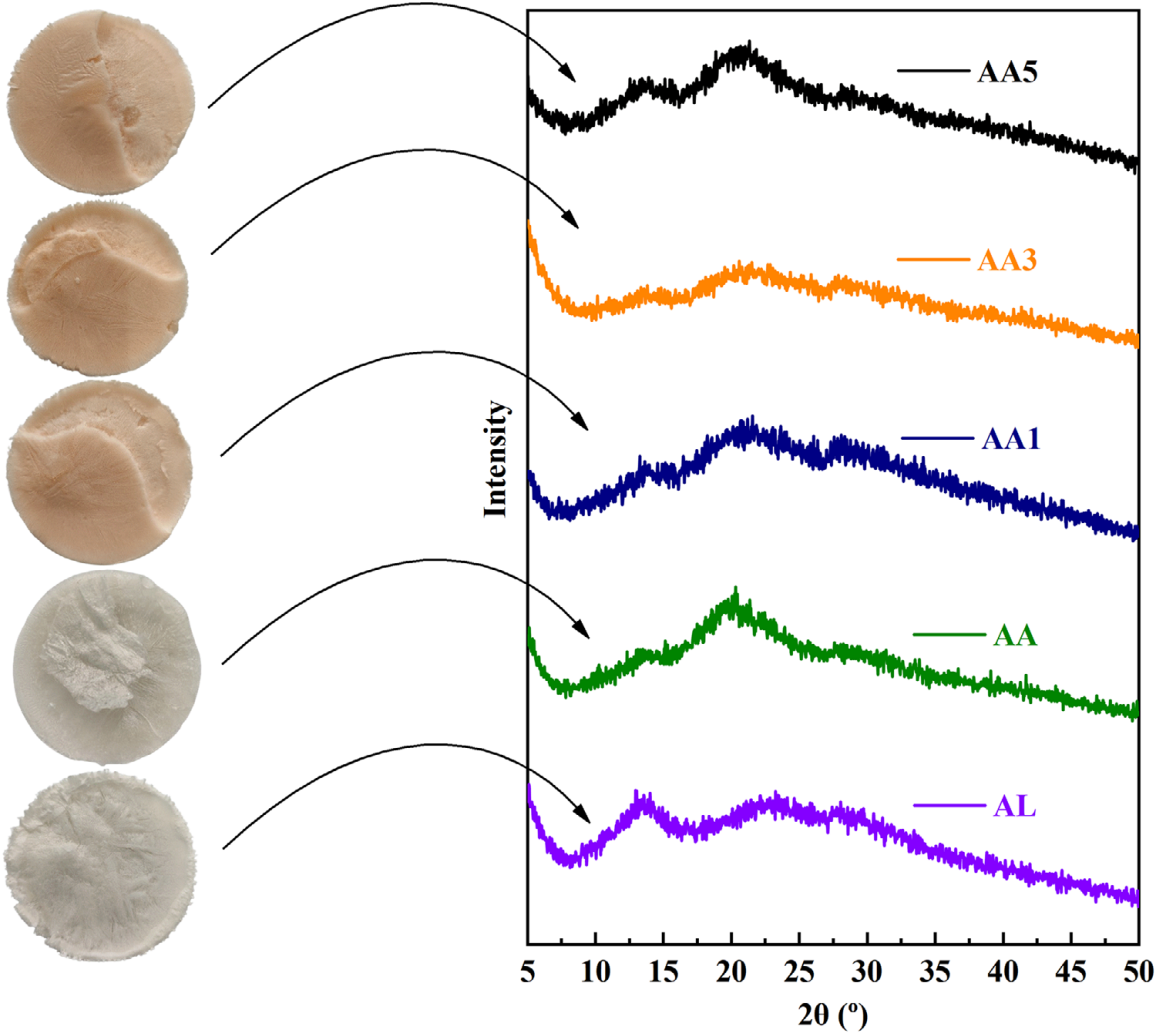
Images (left) of the synthesized sponges and the corresponding XRD patterns (right) in the angular region of 2θ = 5–50°.

Five different sponges were obtained, as described in Table 1, and their XRD analysis is shown in Figure 1. XRD analysis of the freeze‐dried sponges (AL, AA, AA1, AA3, and AA5) showed highly similar profiles in the 2θ range of 5–50°, characterized by broad, low‐intensity halos, indicating the predominantly amorphous nature of all sponges (Figure 1). Broad, diffuse contributions were observed at approximately 2θ = 12–14°, 20–23°, and 28–31°, with only slight differences in intensity and band shape among the sponges. These results suggest that the incorporation of *andiroba* oil and increasing concentrations of the complex did not promote expressive structural rearrangements in the alginate matrix. However, subtle variations may be associated with weak intermolecular interactions among the formulation components [32]. Souza Junior et al. (2026) [30] described similar XRD behavior for sodium alginate‐based sponges containing *andiroba* oil, reporting closely related diffraction profiles among the formulations and reinforcing the predominance of an amorphous structural pattern in these freeze‐dried sponges.

**TABLE 1:** Composition of the developed sodium alginate sponges.

Sponges	Sodium alginate % (*m/v*)	Andiroba oil % (*m/m*)	[Mn(phen)_2_Cl_2_] % (*m/m* ^a^)
AL	2	—	—
AA	2	2	—
AA1	2	2	1
AA3	2	2	3
AA5	2	2	5

^a^ With respect to the mass of sodium alginate.

The structural characteristics of the developed sponges suggest their potential use as topical delivery systems for the incorporated bioactive compounds. Recent studies have demonstrated that alginate‐based sponges can act as modified‐release systems for incorporated bioactives. Dos Santos et al. (2024) [33] reported a curcumin release of 67.9 ± 0.6% in 24 h, from alginate sponges containing κ‐carrageenan beads, with Higuchi kinetics providing the best fit. Aykaç et al. (2025) [34] showed that ibuprofen‐loaded sodium alginate sponges followed first‐order or Hopfenberg release behavior. These reports, together with recent findings on *andiroba* oil‐loaded alginate sponges [30], support the view that the amorphous and porous architecture of the present formulations is compatible with topical delivery applications.

**FIGURE 2:**
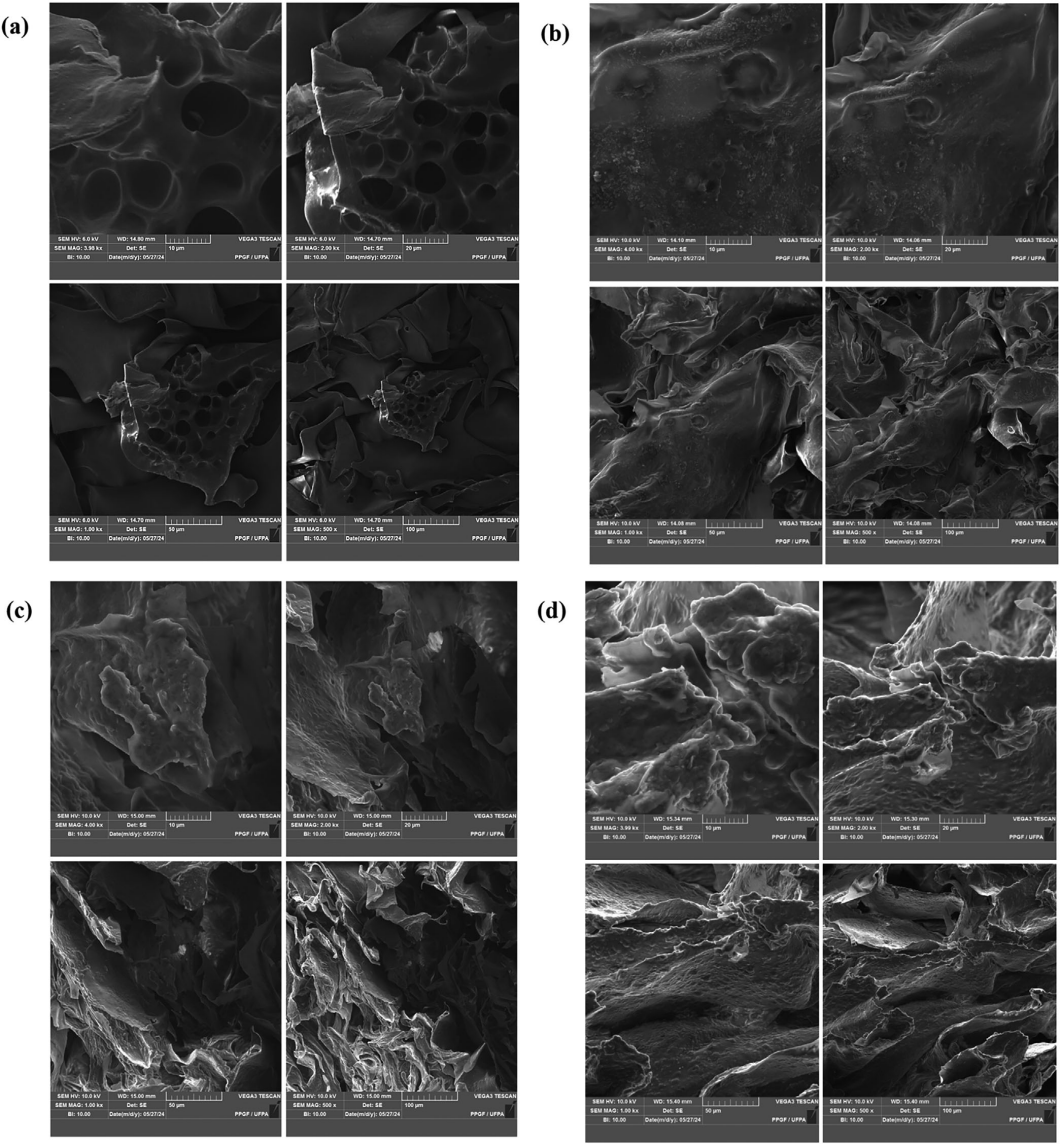
SEM images captured for the sponges: (a) AL; (b) AA1; (c) AA3; and (d) AA5.

The micrographs depicting the morphology of the prepared sponges are seen in Figure 2a–d. The AL sponge shows pores of different sizes and shapes (Figure 2a), while AA1, AA3 and AA5 sponges show leaf‐like structures and an irregular porous morphology (Figure 2b–d). This is attributed to the free space created after removing water by freeze‐drying the sponges embedding both the complex and the oil. According to Sultana et al. (2021) [26], the morphological components of biomaterials, and their pore width, offer broad indications on biochemical stability, cell biocompatibility, waste absorption around the site of use, decomposition, and delivery of drugs over the desired therapeutic period.

SEM image analysis suggests that AL exhibited the highest apparent surface porosity. In contrast, the formulations containing *andiroba* oil and [Mn(phen)_2_Cl_2_] complex showed lower apparent pore area fractions and a more irregular lamellar morphology. Quantitative image analysis using ImageJ confirmed this trend, revealing a surface pore area fraction of 67.2% for AL, compared to 24.5%, 36.3%, and 33.0% for AA1, AA3, and AA5, respectively. These data indicate that the incorporation of *andiroba* oil and [Mn(phen)_2_Cl_2_] complex alters the surface architecture of the alginate sponges, resulting in a less open, more heterogeneous porous structure.

Mechanical performance is an important parameter for alginate‐based wound dressings, since these materials must combine porosity and fluid uptake with sufficient structural integrity for handling and application. Recent studies have shown that alginate sponges can achieve relevant mechanical properties depending on composition [34, 35]. For example, Qi et al. (2024) [35] reported that an amoxicillin‐loaded sodium alginate/cellulose nanocrystals/polyvinyl alcohol composite sponge presented a tensile strength of 1.79 MPa, together with high porosity (84.2%) and flexibility, supporting its applicability as a wound dressing. Aykaç et al. (2025) [34] also described ibuprofen‐loaded sodium alginate sponges and included characterization of elasticity, as measured by texture analysis, among the key parameters for topical use, reinforcing the importance of mechanical evaluation in this class of biomaterials. More broadly, a recent review of alginate wound dressings emphasized that suitable mechanical properties are among the core requirements for clinical performance, along with exudate management and biocompatibility [36].

### 3.2 FT–IR Spectroscopy Studies

**FIGURE 3:**
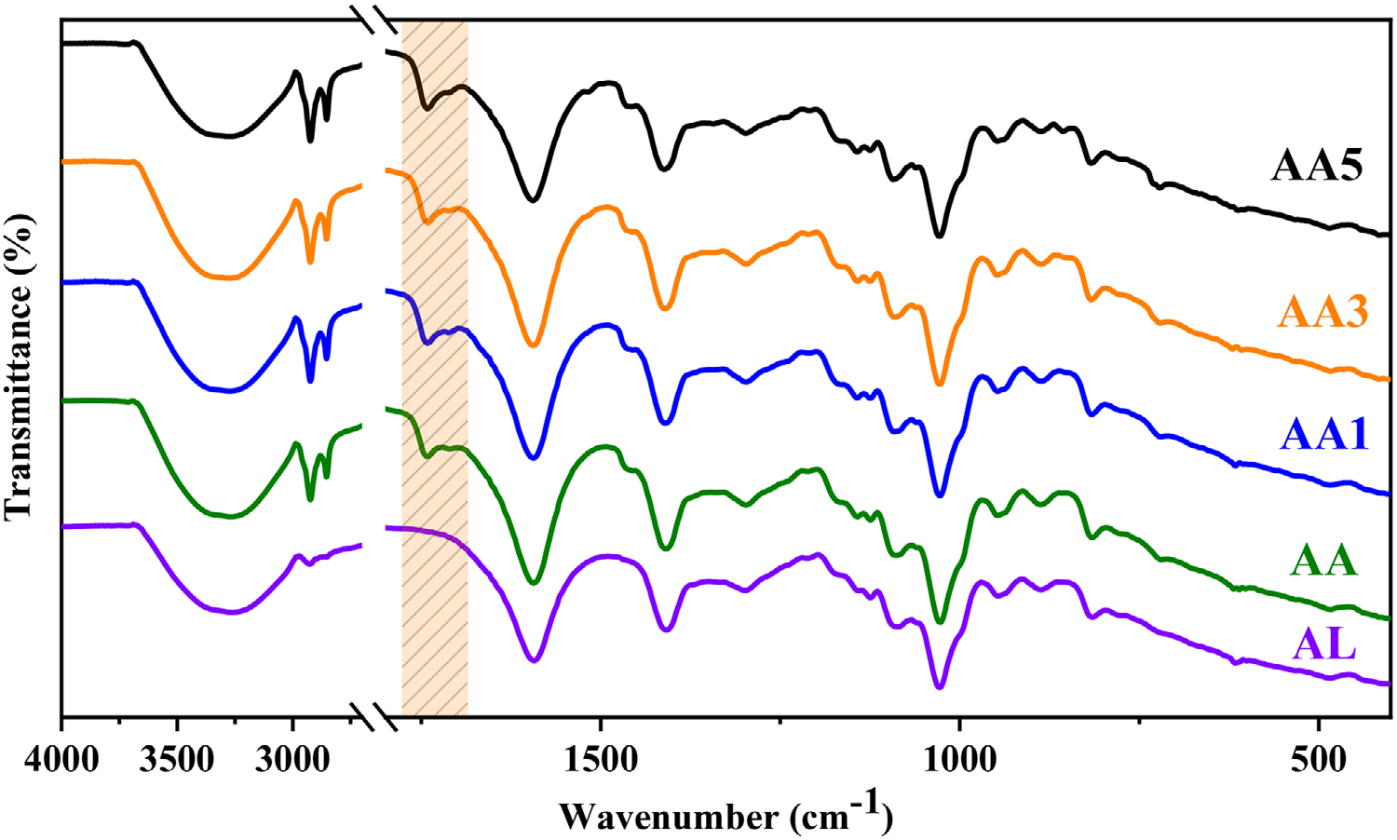
Experimental IR spectra of the sponges. Alginate (purple); alginate and *andiroba* (green); alginate, *andiroba*, 1% concentration of Mn(II) complex (blue); alginate, *andiroba*, 3% concentration of Mn(II) complex (orange) and alginate, *andiroba*, 5% concentration of Mn(II) complex (black).

The IR absorption spectra of the obtained sponges are shown in Figure 3. As presented, bands near 3300–3500 cm^−1^ indicate that the sponges contain numerous OH groups [37]. Modes at about 2922, 1092, and 1408 cm^−1^ were assigned as stretching vibrations of C─H, C─O, and the in‐plane bending vibration of CH─OH, respectively; while the modes at about 1592 and 1031 cm^−1^ were assigned as stretching vibration of C═C and C─O of AL [32].

After adding *andiroba* oil, the spectra of AA, AA1, AA3, and AA5 exhibited similar characteristics due to bands related to the carbonyl of fatty acids present in the oil. The presence of bands referring to the stretching of the carbonyl group (C═O) was observed around 1741 and 1712 cm^−1^. Furthermore, intensities of the stretching vibration bands of CH_3_ (2922 cm^−1^) and CH_2_ (2854 cm^−1^) increased by introducing the oil in the composition of the polymeric matrix of sponges, as pictured in Figure 3.

### 3.3 Thermal Analyses

**FIGURE 4:**
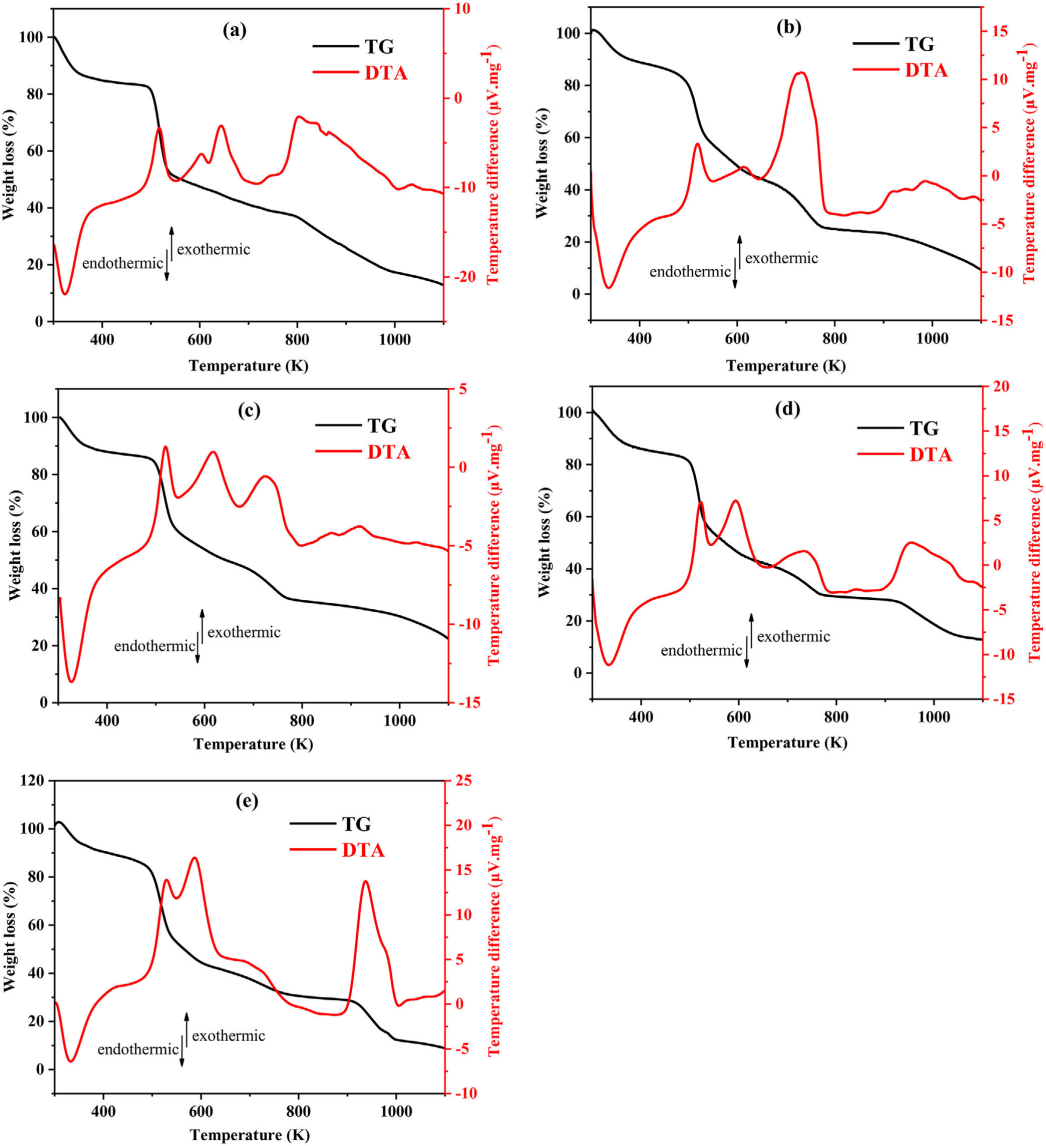
Thermal analyses by TG and DTA of the sponges: (a) AL, (b) AA, (c) AA1, (d) AA3, and (e) AA5.

Figure 4a–e shows the thermal behavior of AL, AA, AA1, AA3, and AA5 sponges through TG and DTA curves. The first event of the five thermograms corresponds to surface dehydration in different temperature ranges, namely, 311–348 K (AL), 313–348 K (AA), 311–348 K (AA1), 310–355 K (AA3), and 311–350 K (AA5). This thermal event is characterized by an endothermic peak in the DTA curve related to residual water removal on the sponges’ surface and clarified by the TG curve. Since water molecules are linked to other compounds through weak bonds, evaporation requires low heat [38].

Partial decomposition of the polymeric material begins in the three events ahead, also in different temperature ranges, namely, 504–529 K (AL), 498–530 K (AA), 504–531 K (AA1), 501–529 K (AA3), and 502–534 K (AA5). Those events are exothermic and involve the breaking of the AL component's polymer chains, and the organic material belonging to the complex and oil incorporated, resulting in significant mass losses, namely, 35.52% in AL (−0.602 mg), 37.10% in AA (−1.820 mg), 31.88% (−1.172 mg) in AA1, 35.65% (−1.677 mg) in AA3, and 49.62% in AA5 (−1.179 mg), due to thermal degradation of the materials.

Furthermore, the sharp drop in the TG curve is associated with the degradation of the polymer structural groups. Discontinuity in this mass loss is observed in the TG curves, which is explained by the decomposition of 1,10‐phenanthroline and the fatty acids present in *andiroba* oil that occur simultaneously with the degradation of the polymer. Additionally, it can be noted that the higher the concentration of the Mn(II) complex, the greater the amount of material remaining in the sample. This fact can be explained by the presence of the Mn(II) ion in the sponges, since it does not undergo decomposition and remains after heat treatment, with only a small loss of mass characteristic of the partial oxidation of the transition metal [31, 38].

**FIGURE 5:**
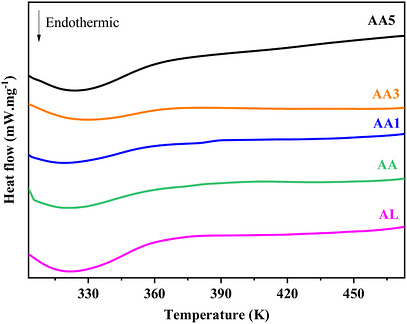
DSC curves of AL, AA, AA1, AA3, and AA5 sponges measured between 300 and 500 K (5 K/min) using N_2_ gas with a 100 mL/min flow rate.

Figure 5 shows the DSC curves of the sponges, indicating an endothermic event related to dehydration as described above for the TG‐DTA. The enthalpies (ΔH) calculated from the dehydration were 43.60 (AL), 45.28 (AA), 46.30 (AA1), 46.42 (AA3), and 46.88 kJ/mol (AA5). The dehydration of each water molecule in the sponges demonstrates a value close to the heat of vaporization of water, which is approximately 41 kJ/mol [39]. Furthermore, the large difference observed for sponges AA, AA1, AA3, and AA5 is attributed to the incorporation of *andiroba oil* into the alginate matrix, which may increase intermolecular contact between the complex and the oil present in the polymeric matrix.

The TG/DTA and DSC results allowed a consistent comparative interpretation of the main thermal events of the developed sponges, particularly the initial dehydration stage and the subsequent decomposition of the organic polymeric matrix. The differences observed among the formulations indicate that the incorporation of *andiroba* oil and the [Mn(phen)_2_Cl_2_] complex affected the thermal behavior of the alginate‐based matrices, especially in the temperature intervals associated with water loss and degradation of the organic components. Similar behavior has been reported for other alginate‐based systems [40, 41, 42], in which the thermal profile is influenced by matrix composition and by the presence of incorporated inorganic and/or organic phases. In particular, recent non‐isothermal thermogravimetric studies have shown that alginate films may exhibit an average activation energy for degradation of about 225 kJ/mol when evaluated by the KAS method [40]. In contrast, Li et al. (2010) [41] described that sodium alginate pyrolysis has also been reported to have an average apparent activation energy of 188.1 kJ/mol. Kragović et al. (2016) [42] described that in composite systems, this parameter may decrease markedly depending on the incorporated phase; for example, the activation energy was reported to decrease from 87.30 kJ/mol for pure alginate to 72.75 and 63.35 kJ/mol in alginate–zeolite composites. The data reinforce that activation energy is highly sensitive to formulation and intermolecular interactions and, therefore, could further refine the discussion of the thermal stability of the present sponges. However, because reliable determination of activation energy requires non‐isothermal analysis performed at multiple heating rates, this kinetic approach was beyond the scope of the present study.

### 3.4 Antibacterial Activity

The disk diffusion test was used to evaluate the antibacterial properties of the different types of freeze‐dried sponges against Gram‐positive (*S. aureus* and *E. faecalis*) and Gram‐negative (*P. aeruginosa* and *E. coli*) bacteria. It can be seen from Table 2 that the diameter of the inhibition zone increased by changing the concentration of [Mn(phen)_2_Cl_2_] complex in the sponges. To compare the release profile and antibacterial effect of the complex with alginate and *andiroba* oil, sponges were analyzed without the [Mn(phen)_2_Cl_2_] complex. The result showed a gradual improvement of the inhibition phenomenon with increased complexity in the sponges. The disc showed that neither the alginate (AL) nor the alginate containing oil (AA) had any antibacterial effect [43]. Even though *andiroba* oil does not have an antibacterial effect at the concentration used (2%, m/m), it is expected that the binding of *andiroba* oil to the complex ensures the enhancement of the bactericidal effect on the tested strains. Thus, changing the concentration of the complex possibly improved the antibacterial effect compared to the sponge without the complex.

**TABLE 2:** Diameter of the zone of the inhibition circle was exhibited by different types of freeze‐dried sponges and positive control (gentamicin).

	Inhibition zone (mm/mg)					
Gram positive strains	AL	AA	AA1	AA3	AA5	Gentamicin
*S. aureus*	0	0	3.67 ± 1.0	11.97 ± 1.0	12.43 ± 3.0	10.67 ± 1.0
*E. faecalis*	0	0	0	12.62 ± 2.4	15.14 ± 1.0	10.67 ± 1.0
Gram negative strains	AL	AA	AA1	AA3	AA5	Gentamicin
*P. aeruginosa*	0	0	0	12.38 ± 2.2	16.11 ± 2.3	10.67 ± 1.0
*E. coli*	0	0	0	19.50 ± 1.2	24.03 ± 1.5	10.67 ± 1.0

Sultana et al. (2021) [26] tested the sponges on a single colony of Gram‐positive bacteria, *S. aureus* (ATCC 6538), and Gram‐negative bacteria, *E. coli* (ATCC 8739). The results showed an antibacterial effect on both colonies tested, as evidenced by the formation of inhibition zones, demonstrating the sponge's antibacterial efficacy. Lan et al. (2015) [44] also reported bacteriostatic effects against *E. coli* and *S. aureus*.

Our study demonstrated satisfactory results against all strains used. Yet the sponges showed better bactericidal effects against Gram‐negative bacteria than against Gram‐positive bacteria. One of the microorganisms commonly causing wound infections is *E. coli* [45], against which the sponge inherently shows antibacterial efficacy, and its subsequent development will reduce the disease burden caused by infection by such microorganisms.

In this experiment, the commercial antibiotic gentamicin was used as a positive control group. This antibiotic is effective against a variety of bacterial infections [46, 47]. The antibacterial efficacy of the sponge containing the complex and the oil was higher than that of the drug used as a positive control in this study. This could be due to the efficient incorporation of the Mn(II) complex and *andiroba* oil in the polymeric matrix, resulting in an ideal control of the release of these bioactive compounds, allowing optimization of the therapeutic potential in various dressings. In addition, the subsequent development of this sponge could have great potential to reduce the burden of disease caused by microbial infections.

The antibacterial performance of the developed sponges should be interpreted in the context of the biological properties of both incorporated components. Previous work [31] demonstrated that the isolated [Mn(phen)_2_Cl_2_] complex exhibits measurable antibacterial potency, with MIC values of 125 µg/mL against *S. aureus*, 15.12 µg/mL against *E. faecalis*, 7.81 µg/mL against *E. coli*, and 31.25 µg/mL against *P. aeruginosa*, showing bactericidal action against *S. aureus*, *E. faecalis*, and *P. aeruginosa*, and bacteriostatic behavior against *E. coli*. In contrast, *andiroba oil* has been reported to display more moderate direct antibacterial activity, with MIC values of 400 µg/mL against *S. aureus* and *E. faecalis* [48]. At the same time, its relevance in this system extends beyond isolated antimicrobial potency. In a recent study [30], sodium alginate sponges containing *andiroba* oil significantly reduced nitric oxide production in LPS‐activated RAW 264.7 macrophages at 250 and 500 µg/mL, while maintaining cell viability above 90% across the 50–500 µg/mL concentration range, confirming anti‐inflammatory activity and low cytotoxicity. Therefore, the biological response of the developed sponges may arise from the combined effect (anti‐inflammatory and antibacterial) of the complex *andiroba* oil, which together reinforce the potential of this biomaterial for topical wound‐related applications.

## 4 Conclusions

Freeze‐dried sodium alginate sponges containing *andiroba* oil and the [Mn(phen)_2_Cl_2_] complex were successfully developed as porous biomaterials for topical applications. XRD analysis showed that all sponges exhibited a predominantly amorphous profile. At the same time, SEM revealed that incorporation of *andiroba oil* and complex altered the surface architecture of the alginate matrix, reducing the apparent surface pore area fraction from 67.2% in the unloaded sponge (AL) to 24.5%, 36.3%, and 33.0% in AA1, AA3, and AA5, respectively, and promoting a more irregular lamellar morphology. FT–IR spectra confirmed the characteristic functional groups of alginate and *andiroba* oil, especially hydroxyl, carbonyl, and carboxylate‐related bands, supporting the presence of the oil within the polymeric dressing. Thermal analyses demonstrated an initial dehydration event followed by decomposition of the organic polymeric matrix, with sponge‐dependent differences associated with the incorporated components. Antibacterial evaluation showed that the sponges containing higher [Mn(phen)_2_Cl_2_] loadings, particularly AA3 and AA5, exhibited the most pronounced inhibitory activity against the tested bacteria. AA3 and AA5 reached inhibition zones of 11.97 ± 1.0 and 12.43 ± 3.0 mm/mg against *S. aureus*, 12.38 ± 2.2 and 16.11 ± 2.3 mm/mg against *P. aeruginosa*, 12.62 ± 2.4 and 15.14 ± 1.0 mm mg^−^^1^ against *E. faecalis*, and 19.50 ± 1.2 and 24.03 ± 1.5 mm/mg against *E. coli*, respectively. These results indicate that increasing the loading of complex improved the sponges’ antibacterial response. When considered together with our previous findings reported for the isolated [Mn(phen)_2_Cl_2_] complex and for *andiroba* oil‐loaded alginate sponges, the present data support the view that this biomaterial combines antibacterial performance with the biological relevance of *andiroba* oil as a multifunctional component. Overall, the developed sponges represent promising alginate‐based biomaterials for topical wound‐related applications; studies on mechanical performance to determine the elasticity, resistance, and texture are ongoing, together with the biocompatibility assessment.

## Author Contributions

All authors actively contributed to the conceptualization, design of the study, methodology, validation, formal analysis, investigation, data curation and validation, literature review, software management, resources and facilities, writing – original draft preparation, writing – review and editing, project administration, supervision, and funding acquisition. All authors have made a substantial contribution to the work.

## Use of Generative AI and AI‐Assisted Technologies in the Writing Process

No artificial intelligence (AI) or AI‐assisted technologies were used in the writing process of this manuscript.

## Ethics Statement

The authors have nothing to report.

## Consent

All authors agreed with the final version of this manuscript and with the current submission.

## Conflicts of Interest

The authors declare no conflicts of interest.

## Data Availability

Data are available from the authors upon request.
